# From Philanthropy to Clinical Care through Research: Impact of the Norman Saunders Complex Care Initiative

**DOI:** 10.3390/children9030395

**Published:** 2022-03-11

**Authors:** Colin Macarthur, Eyal Cohen, Sherri Adams, Francine Buchanan, Natasha R. Saunders, Jeremy N. Friedman

**Affiliations:** 1 Child Health Evaluative Sciences, Hospital for Sick Children Research Institute, Peter Gilgan Centre for Research and Learning, 686 Bay Street, Toronto, ON M5G 0A4, Canada; eyal.cohen@sickkids.ca (E.C.); sherri.adams@sickkids.ca (S.A.); francine.buchanan@sickkids.ca (F.B.); natasha.saunders@sickkids.ca (N.R.S.); jeremy.friedman@sickkids.ca (J.N.F.); 2 Department of Paediatrics, University of Toronto, Toronto, ON M5S 1A1, Canada; 3 Institute of Health Policy, Management and Evaluation, University of Toronto, Toronto, ON M5S 1A1, Canada; 4 Complex Care Program, Hospital for Sick Children, Toronto, ON M5G 1X8, Canada; 5 Lawrence S Bloomberg Faculty of Nursing, University of Toronto, Toronto, ON M5S 1A1, Canada

**Keywords:** children with medical complexity, complex care, research, evaluation

## Abstract

Norman Saunders was a respected academic community paediatrician who was passionate about the care of children with medical complexity. Following his untimely death at age 60, patients, friends, and colleagues raised funds to create the Norman Saunders Complex Care Initiative (NSCCI). Dr. Saunders’s vision was a comprehensive, coordinated, and integrated clinical program for children with medical complexity that was informed by research evidence. The objective of this review was to evaluate the impact of targeted philanthropic funding on research, clinical care, and policy. Since 2006, NSCCI funds have been used to support interdisciplinary and innovative research. Funded projects have reflected a breadth of research questions (clinical care, training, health system delivery, social determinants), disciplines, and methods, and the research results have informed and helped build an internationally renowned clinical program in complex care. Philanthropic funding was the foundation for the NSCCI, which over the last 15 years has built research and clinical capacity, catalysed clinical and research networks, helped train paediatric residents, influenced policy, and improved the health and well-being of children with medical complexity and their families across Canada, and beyond.

## 1. Introduction

Norman (Norm) Saunders was an academic community paediatrician in Toronto, with a thriving primary care practice, and an Associate Professor appointment in the Department of Paediatrics at the University of Toronto. Norm also provided in-patient care and taught paediatric residents at the Hospital for Sick Children as a member of the Division of Paediatric Medicine. In the language of the time, Norm was an authentic “triple threat”—a well-respected clinician with a keen interest in research and teaching. Norm’s academic outputs included peer-reviewed publications, books on parenting, and clinical teaching awards [1,2,3,4]. 

A serious illness led to Norm’s premature death at 60 years of age. In the final year of his life, patients, friends, and colleagues raised funds for a philanthropic donation in his name. Despite the knowledge that he was terminally ill, Norm participated with humility, grace, and enthusiasm in the discussions about possible initiatives. It was Norm’s desire that the funds be used to make a difference in the care, health, and well-being of children with medical complexity (CMC) and their families. Norm’s clinical experience in community practice and on the wards at the Hospital for Sick Children in Toronto had led him to advocate for better care and policy for these vulnerable children and their families. He had seen first-hand that the lack of care coordination was causing poor health outcomes for the children as well as increased stress for families trying to navigate a fragmented system. Comprehensive, coordinated, and integrated clinical care of CMC was the goal, however, a research focus for the initiative was also essential, given Norm’s belief in evidence-informed care as the foundation for healthcare improvement. Ahead of his time, Norm advocated for ‘patient-oriented research’ in CMC, i.e., research that met patient needs and was therefore meaningful and relevant for patients and families. For example, Norm was concerned about the lack of meaningful outcomes (research and clinical) in complex care and how best to measure these outcomes. The philanthropic goal was to create a meaningful, fitting, and long-lasting legacy for a respected colleague and clinician. The purpose of this commentary is to review the impact of targeted philanthropic funding on research, clinical care, and policy in the area of CMC.

Children and youth with medical complexity have been defined as those with complex chronic conditions and/or neurologic impairment who have substantial healthcare needs, functional limitations, require specialised care, and have high health resource utilisation [5]. Less than 1% of children and youth in Canada meet this definition; however, the impact on the health system is significant—children and youth with medical complexity account for 33% of total paediatric healthcare expenditures, 37% of hospital admissions, and 54% of total hospital days in Canada [6].

## 2. Materials and Methods

The Hospital for Sick Children Foundation launched the Norman Saunders Complex Care Initiative (NSCCI) in 2006. Through word-of-mouth alone, donations from colleagues, friends, and family raised $250,000 to fund the initial set up of a peer-reviewed, open competition grants program. The endowment has subsequently grown to almost CAD 2 million.

The impact of the NSCCI initiative was determined through document review, literature review, and discussion with key clinical leaders. Documents reviewed included terms of reference for the NSCCI, mission and vision statements, grant review processes, grant competition results, end-of-grant reports, and annual Foundation reports. A PubMed search was used to identify published papers resulting from the successful NSCCI grant applications. Discussions with key clinical leaders provided information on the complex care clinical program. The impact of the NSCCI was evaluated on three domains: research, clinical care, and policy. The time frame for review was from the launch of the NSCCI in 2006 to the end of 2021.

## 3. Results

### 3.1. The NSCCI Grants Competition

The purpose of the NSCCI Grants Competition is to promote interdisciplinary and innovative research related to complex care in child health. Applications must be focused on children with medical complexity, defined as children with (i) one or more complex chronic conditions that are multi-system and severe, (ii) significant functional limitation, often leading to reliance on technology, e.g., feeding tubes, (iii) high healthcare utilisation, requiring specialised care/services across providers and sectors, and (iv) a need for care provision in the home as well as care coordination. Researchers (in particular, new or early career) and healthcare professionals affiliated with an academic health sciences centre and/or University are encouraged to apply. Since 2019, all NSCCI applications must include a plan for partnership with patients and families as research partners and include patient engagement costs in the budget. 

The NSCCI grant competition is national in scope, held annually in the fall, and typically funds two to three proposals to a maximum of CAD 50,000 per proposal. The grant review panel is composed of healthcare professionals (paediatricians, nurses, social workers) and researchers. In 2019, a parent of a child with medical complexity joined the panel. Each application is reviewed by two panel members to determine relevance to complex care, quality of study methods, the expertise of the research team, and comprehensiveness of the patient engagement plan. The parent member of the panel reviews all applications and provides specific feedback on the family burden of research methods, the relevance of the research to the family experience, and the patient engagement plan. The first NSCCI grants competition took place in 2008. For the first decade, the competition was local (applicants required an affiliation with a Toronto University); in 2019, the competition became national in scope. Grants are typically one year in duration and all successful applicants are required to provide an end-of-grant final report.

### 3.2. Impact of the NSCCI on Research

Over 14 years (2008–2021 inclusive), the NSCCI Grants Competition has funded 41 research projects, with more than CAD 1 million allocated to research focused on CMC and their families. As would be expected, given the breadth of the clinical population, the funded projects also reflect a breadth of research questions, disciplines, and methods. Table 1 highlights a selection of projects funded through the NSCCI grants competition whose results have subsequently been published in high-quality, peer-reviewed clinical journals [7,8,9,10,11,12,13,14,15,16,17].

Almost two-thirds of the NSCCI-funded projects (27/41) have focused on clinical care. These projects span the entirety of clinical care, with studies on diagnostic testing (genome sequencing), treatment (surgical interventions), care planning (care plans and advanced care planning), clinical decision-making (caregiver approaches to shared decision-making), measurement (core outcome sets), and end-of-life care [7,8,9,10,11,12]. Education was the focus for 3/41 grants. For example, a mixed methods study (randomised controlled trial and qualitative interview) examined the competency of paediatric residents in (simulated) tracheostomy care and their perceived self-efficacy in caring for CMC following completion of an educational curriculum [13]. Health service delivery for CMC was the focus for 6/41 grants. Examples include the evaluation of an integrated complex care model and the evaluation of a remote satellite CMC clinic [14]. Social determinants of health in the context of CMC and their families were addressed in 5/41 studies. For example, two projects focused on housing, evaluating residential movement patterns and the housing needs of families of CMC [15,16]. Last, as an example of innovation funded through the NSCCI grants competition, an engineering-based study examined the impact of music generated from physiological signals (“biosongs”) on caregiver perceptions of their interactions with individuals with communication challenges because of multiple profound disabilities [17].

From a capacity-building perspective, the initiative has been a tremendous success. In total, 44 PIs/co-PIs from across Canada, along with 110 co-investigators on research teams, have been funded. In other words, the NSCCI has supported more than 150 different individuals—researchers, healthcare professionals, trainees, and parents—from across Canada to conduct research on the health and well-being of CMC and their families. A direct result of the funding is that the NSCCI grants competition has helped build an informal network of researchers, as well as a community of research practice that spans disciplines (medicine, surgery, social sciences, engineering, education) and research methods (from qualitative research to randomised controlled trials). 

### 3.3. Impact of the NSCCI on Clinical Care

The development of a ‘Complex Care Program’ was the clinical component of the NSCCI, and the impact of the initiative on clinical care has been remarkable. At the launch of the initiative, only a few families were cared for at the Hospital for Sick Children, clinical care was fragmented, health and well-being outcomes were largely undefined, and there was a dearth of clinical expertise. Fourteen years later, the Complex Care Program at the Hospital for Sick Children is an international model of success.

The clinical program launched in 2006, with leadership from the Division of Paediatric Medicine. The on-site clinic initially cared for a few dozen patients and focused mainly on outpatient care. Research findings from early NSCCI projects that evaluated complex care programs, however, identified the need for community collaboration and families’ desire for a shift in the location of care provision from the hospital to closer to the family home. As a result, partnerships with community organisations were developed. NSCCI projects also conducted process and outcome evaluations of integrated care models. These research findings underpinned the rapid expansion of the Complex Care Program which now serves 550 children with medical complexity at the Hospital for Sick Children and 10 community-based satellite clinics and serves as an exemplar model of care delivery for this population of children and their families internationally. 

In essence, the program evaluation studies funded by the NSCCI have provided the relevant evidence to inform and develop a coordinated, comprehensive, and collaborative complex care program that meets the needs of CMC and their families across the province. Evidence generated by the NSCCI-funded projects has led to an Ontario-wide clinical service delivery model—‘Complex Care for Ontario’—funded by the Ontario Ministry of Health. In addition, numerous international delegations from around the world (USA, Europe, Asia, Australasia) have visited the Hospital for Sick Children to learn about the complex care program and implement the model in their home countries. This speaks to the credibility and innovative nature of the clinical program that has been built on research evidence.

NSCCI-funded projects on diagnosis, treatment, and prognosis have been used to develop evidence-informed care plans for CMC that have been disseminated and implemented internationally. The findings from NSCCI projects on resident training in complex care have been incorporated into the teaching curriculum for paediatric residents. Last, the results of the social determinants of health research funded by NSCCI have been used in social policy decision-making by relevant provincial government departments.

### 3.4. Future Plans and Reflections

The Hospital for Sick Children Foundation has carefully stewarded the philanthropic funds donated to support the NSCCI initiative. As of 2022, the NSCCI endowment has grown to almost CAD 2 million and the endowment funds will continue to support the NSCCI national open grants competition that funds two to three operating grants on CMC each year. 

Several factors have likely contributed to the success of the NSCCI initiative. First, the Hospital for Sick Children Foundation has worked diligently to raise funds for the initiative and steward the donations. Second, the grants competition has led to high-quality, relevant research because of rigorous peer-review and panel discussion and the requirement for patient partners in all applications. Third, the clinical CMC program at the Hospital for Sick Children has succeeded because of strong institutional support at the divisional and departmental levels. Last, the NSCCI, and CMC clinical leaders have built strong relationships with knowledge users and policymakers, which has led to the translation and implementation of NSCCI research findings into practice and policy provincially, nationally, and internationally.

## 4. Discussion

Philanthropic donations to honour the memory of Dr. Saunders led to the development of the NSCCI. Norm’s vision was that children with CMC (and their families) would receive comprehensive, coordinated, integrated, high-quality clinical care, based on high-quality research evidence. The NSCCI grants competition has generated a substantial body of research evidence on complex care; a clinical area that has traditionally been under-researched. Further, the findings from NSCCI projects have directly and significantly influenced clinical care, education, and policy for CMC and their families. The NSCCI initiative has raised awareness of the challenges faced by CMC and their families, built research and clinical capacity, catalysed clinical and research networks, helped train paediatric residents and allied health professionals (nurse practitioners, dieticians), and improved the health and well-being of children with medical complexity and their families across Canada and beyond. Norm would be proud.

## Figures and Tables

**Table 1:** Characteristics of selected publications resulting from NSCCI-funded projects.

First Author [Ref]	Title of Published Paper	Study Design	Journal, Year (Impact Factor, # Citations *)
Costain [7]	Genome sequencing as a diagnostic test in children with unexplained medical complexity.	Prospective cohort of families (single centre)	*JAMA Network Open*, 2020 (IF 8.483, 10 citations)
Nelson [8]	Survival and surgical interventions for children with trisomy 13 and 18.	Retrospective cohort (single centre)	*JAMA,* 2016 (IF 56.272, 132 citations)
Adams [9]	Exploring the usefulness of comprehensive care plans for children with medical complexity (CMC): A qualitative study	Qualitative interview of parents and HCPs	*BMC Pediatrics* (IF 2.030, 71 citations)
Orkin [10]	Toward an understanding of advance care planning in children with medical complexity.	Qualitative interview of parents and HCPs	*Pediatrics*, 2020 (IF 7.124, 18 citations)
Joachim [11]	Core outcome set for children with neurological impairment and tube feeding.	Consensus: caregivers, HCPs, and researchers	*Dev Med Child Neurol,* 2020 (IF 5.449, 7 citations)
Rapoport [12]	Parental perceptions of forgoing artificial nutrition and hydration during end-of-life care.	Qualitative interview of parents	*Pediatrics,* 2013 (IF 7.124, 53 citations)
Huth [13]	Evaluating curricular models in the care of children with medical complexity: a mixed methods randomized controlled trial.	Mixed methods: 2-site randomised trial and qualitative interview	*Academic Pediatrics,* 2020 (IF 3.995, 2 citations)
Cohen [14]	Integrated complex care model: lessons learned from inter-organizational partnership.	Process evaluation of complex care model	*Healthcare Quarterly,* 2011 (IF 0.554, 25 citations)
Esser [15]	Housing need among children with medical complexity: a cross-sectional descriptive study of three populations.	Cross-sectional survey of caregivers	*Academic Pediatrics,* 2021 (IF 3.995, 1 citation)
Cohen [16]	Residential movement patterns of families of young children with chronic conditions in Ontario, Canada.	Population-based cohort (administrative health data)	*Int J Equity Health,* 2013 (IF 3.805, 17 citations)
Blain-Moraes [17]	Biomusic: a novel technology for revealing the personhood of people with profound multiple disabilities.	Sound generation from physiological signals. Caregiver interview	*Augment Altern Comm*, 2013 (IF 2.214, 26 citations)

*: As of January 2022; IF: impact factor; HCP: healthcare professional; #: number of.

## Data Availability

Not applicable.
